# Novel metrics for growth model selection

**DOI:** 10.1186/s12982-018-0072-z

**Published:** 2018-02-23

**Authors:** Matthew R. Grigsby, Junrui Di, Andrew Leroux, Vadim Zipunnikov, Luo Xiao, Ciprian Crainiceanu, William Checkley

**Affiliations:** 1 Division of Pulmonary and Critical Care, School of Medicine, Johns Hopkins University, 1830 E. Monument Street, 5th Floor, Baltimore, MD 21287 USA; 2 Department of Biostatistics, Johns Hopkins Bloomberg School of Public Health, Baltimore, MD USA; 3 Department of Statistics, North Carolina State University, Raleigh, NC USA

## Abstract

**Background:**

Literature surrounding the statistical modeling of childhood growth data involves a diverse set of potential models from which investigators can choose. However, the lack of a comprehensive framework for comparing non-nested models leads to difficulty in assessing model performance. This paper proposes a framework for comparing non-nested growth models using novel metrics of predictive accuracy based on modifications of the mean squared error criteria.

**Methods:**

Three metrics were created: normalized, age-adjusted, and weighted mean squared error (MSE). Predictive performance metrics were used to compare linear mixed effects models and functional regression models. Prediction accuracy was assessed by partitioning the observed data into training and test datasets. This partitioning was constructed to assess prediction accuracy for backward (i.e., early growth), forward (i.e., late growth), in-range, and on new-individuals. Analyses were done with height measurements from 215 Peruvian children with data spanning from near birth to 2 years of age.

**Results:**

Functional models outperformed linear mixed effects models in all scenarios tested. In particular, prediction errors for functional concurrent regression (FCR) and functional principal component analysis models were approximately 6% lower when compared to linear mixed effects models. When we weighted subject-specific MSEs according to subject-specific growth rates during infancy, we found that FCR was the best performer in all scenarios.

**Conclusion:**

With this novel approach, we can quantitatively compare non-nested models and weight subgroups of interest to select the best performing growth model for a particular application or problem at hand.

## Background

Childhood growth modeling plays an important role in understanding and surveilling health outcomes at both individual and population levels. Specific uses include predicting health outcomes based on current trajectories (e.g. failure to thrive, obesity, stunting, wasting) and understanding associations between growth outcomes and childhood exposures (e.g. environmental, gestational, disease) [1, 2]. Many types of statistical approaches have been proposed to model growth measurements as functions of age and related baseline covariates [3–11]. Frequently used statistical models such as linear mixed effects, quantile regression, and functional principal components methods provide great modeling flexibility and are often able to address key features of growth data such as sparsity of sampling, cross-sectional skewness, and smoothness of growth trajectories [12–14].

Comparing models requires an objective criterion that can be uniformly applied to all of them. Nested models can be compared via metrics such as the likelihood ratio test (LRT) or F-test, and penalization for parametrization with the Akaike Information Criterion (AIC) or the Bayesian Information Criterion (BIC). However, comparing non-nested models is complicated because not all models optimize the same objective functions. Therefore, a comprehensive model selection strategy among competing, often non-nested, models necessitates development of a universal selection criterion.

We propose a novel approach based on modifications of the mean squared error, including normalization, age-stratification, and weighting for subject-specific growth rates. These methods differ from those mentioned above in that they measure model predictive performance rather than model fit. Quantifying predictive accuracy at the subpopulation level is critically important in auxology applications. For example, subpopulations representing lower quantiles of growth often contain children who are either stunted or faltering and may require special attention. In such scenarios, model choice may necessarily be driven disproportionately by accuracy of predicting outcomes among said subpopulations. These proposed modifications are centered on an idea of using out-of-sample prediction accuracy as universal measures of model performance.

## Methods

### Study setting

This analysis used data collected in the CONTENT study, located in the two peri-urban communities of Pampas de San Juan Miraflores and Nuevo Paraíso. Both were high density populations located approximately 25 km south of Lima [15]. The original purpose of this study was to examine the impact of Helicobacter pylori on child growth using World Health Organization Multicentre Growth Reference Study standards for calculating height and weight Z scores [15]. Further characterization of these regions can be found in previous publications [15, 16].

### Study design

Data was collected longitudinally between May 2007 and February 2011 [15, 16]. Children were not included if they had severe disease requiring hospitalization, were part of a multiple pregnancy, had a birth weight less than 1500 grams, and/or their parents had intentions of moving during the period of the study [15]. Data was collected at birth with follow up lasting until the age of 24 months. Additional information on study design, including more specific details on information collected, can be found in the original publication [15].

### Biostatistical models

When studying growth-related health outcomes and exposures, height and weight are usually collected at multiple time points to assess individual growth trajectories [1, 4, 17–20]. Notable features of longitudinal data include within-subject correlation, heterogeneity of individual baseline, and dynamic growth [21]. In this study, we employ traditional growth models such as linear mixed effects (LME), as well as less well known techniques such as functional concurrent regression (FCR) and functional principal component analysis (fPCA) [13, 21–24]. For simplicity, we used height as our growth outcome in this study. Let $Y_{ij}$ denote the height of child $i$ at time point $j$, and $t_{ij}$ is the corresponding age for child $i$ at time point $j$, where $i = 1, 2, \ldots , 215$, and $j = 1, 2, \ldots , m_{i}$. Sex effect was included in LME and FCR models, and we denote $X_{i}$ to be the sex for subject $i$. Even though linear regression with truncated cubic splines is well known and simple to implement, Grajeda et al. showed they were inaccurate when modeling longitudinal growth because they did not account for the nature of repeated measurements clustered within subjects and because the assumption on independence between measurements was violated [21].

### Parametric, linear mixed effects model

Inclusion of subject-specific random effects is a convenient way to account for subject level clustering and is easy to implement in most statistical software packages [3, 13, 21, 25].

Since growth exhibits a pronounced non-linear association with age, population mean growth is modeled using truncated cubic splines with knots at 3, 6, 12, and 18 months. Random slopes and intercepts were used to capture the heterogeneity in growth curves. Specifically, random intercepts depict shifts (up or down) of subject-level growth from the population-level intercept, while random slopes represent subject-level growth velocity around the population prediction.

Although standard LME models are intended to account for within subject correlation, it has been shown that, in growth data, random intercept and slope models may have autocorrelated residuals [21]. Therefore, we used a continuous autoregressive error of order one to model the correlation structure between pairs of measurements for any subject. The model is formulated as

$$Y_{ij} = (\beta_{0} + b_{0i} ) + (\beta_{1} t_{ij} + b_{1i} t_{ij} ) + \beta_{2} t_{ij}^{2} + \beta_{3} t_{ij}^{3} + \mathop \sum \limits_{k \in 3,6,12,18} \gamma k\left( {t_{ij} - k} \right)_{ + }^{3} + \alpha_{1} X_{i} + \in_{ij}$$

$$\left[ {\begin{array}{*{20}c} {b_{0i} } \\ {b_{1i} } \\ \end{array} } \right] \sim MVN\left( {\left[ {\begin{array}{*{20}c} 0 \\ 0 \\ \end{array} } \right],\left[ {\begin{array}{*{20}c} {g_{11} } \\ {g_{21} } \\ \end{array} \begin{array}{*{20}c} {g_{12} } \\ {g_{22} } \\ \end{array} } \right]} \right)$$

$$\left[ {\begin{array}{*{20}c} { \in_{i1} } \\ \vdots \\ { \in_{{im_{i} }} } \\ \end{array} } \right] \sim MVN\left( {\left[ {\begin{array}{*{20}c} 0 \\ \vdots \\ 0 \\ \end{array} } \right],\sigma^{2} \left[ {\begin{array}{*{20}c} 1 & \cdots & {\rho^{{\left| {t_{i1} - t_{{im_{i} }} } \right|}} } \\ \vdots & \vdots & \vdots \\ {\rho^{{\left| {t_{i1} - t_{{im_{i} }} } \right|}} } & \cdots & 1 \\ \end{array} } \right]} \right)$$

where $\beta$’s and $\gamma$’s represent the fixed effects of time and age on height, while $b_{0i}$ and $b_{1i}$ represent the random intercepts and slopes, respectively. We assume independence between subjects.

### Nonparametric, functional models

It has been noted that some parametric models may not be sufficiently flexible to fully capture the non-linearity in individual growth trajectories [24]. Therefore, nonparametric approaches have gained popularity in recent years to deal with longitudinal data. One reason to think of repeated measurements as functions at different time points is because the derivatives could be of interest as well (e.g. growth rates of children). Two functional approaches are discussed next.

Functional principal component analysis has become a first-line approach to analyzing functional or longitudinal data [22, 26–29]. It involves non-parametric estimation of the covariance structure and identifying the dominant features (eigenfunctions) of the covariance matrix. Subjects’ random effects are a linear combination of a relatively small number of the eigenfunctions. This allows for increased complexity in the shape of estimated subject-level trajectories, but typically requires more parameters to be estimated than with LME models. Fast Covariance Estimations (FACEs) was developed as a fast bi-variate smoothing method for the covariance operator which has been proved to be widely reliable and computationally efficient [30]. A newer version of FACEs was designed to handle sparse functional data with a revised bivariate smoother, and a fast algorithm for approximating the leave-one-subject-out cross validation for selection of the smoothing parameter [31]. The model can be expressed as

$$Y_{ij} = f_{0} \left( {t_{ij} } \right) + b_{i} \left( {t_{ij} } \right) + \in_{ij}$$

$$\in_{ij} \sim N\left( {0,\sigma^{2} } \right)$$

where $f\left( \cdot \right)$ is a smooth mean function and $b_{i} \left( \cdot \right)$ is generated from a zero-mean Gaussian process with covariance operator $C\left( {s,t} \right) = Cov\left( {b_{i} \left( s \right),b_{i} \left( t \right)} \right)$. Detailed methods to model and estimate $C\left( {s,t} \right)$ as tensor-product splines and to predict subject $i$’s growth curve $X_{i} \left( t \right) = f\left( {t_{i} } \right) + u_{i} \left( {t_{ij} } \right)$ can be found in Xiao et al. [31].

Functional principal component analysis is a way to examine functional variability, however, it is not directly comparable to LME models since it does not take into account effects of other covariates such as gender. As a generalization, we will consider functional concurrent regression (FCR) as a more natural extension of both LME and fPCA because they include time invariant gender fixed effects which correspond with the LME models, but also utilize benefits of modeling growth data as a complex function similar to fPCA. Functional concurrent regression models were introduced and developed in recent years [24, 32–39]. The comparable FCR model to the LME model specified above can be expressed as

$$Y_{ij} = f_{0} \left( {t_{ij} } \right) + \alpha_{1} X_{i} + b_{i} \left( {t_{ij} } \right) + \in_{ij}$$

$$\in_{ij} \sim N\left( {0,\sigma^{2} } \right)$$

where $f_{0} \left( \cdot \right)$ is a smooth estimate of the average population growth curve, $\alpha_{1}$ is the time-invariant fixed sex effect, and $b_{i} \left( \cdot \right)$ models the subject-specific random functional deviation of subject $i$ and is generated from a zero-mean Gaussian process with covariance function $C\left( {s,t} \right)$. Furthermore, $b_{i} \left( \cdot \right)$ and $( \in_{i1} , \ldots , \in_{{im_{i} }} )$ are assumed to be mutually independent across subjects. Smoothing parameters can be selected using either restricted maximum likelihood or generalized cross validation as described by Wood et al. [40, 41]. From a modelling perspective, it is notable that fPCA is a special case of FCR without effects from covariates other than time. The addition of fixed effects in this context is non-trivial. Details on the FCR estimation procedure are further described by Leroux et al. and an accompanied R package [42, 43].

### Definition of comparison criteria metrics

In this section, we introduce three metrics to perform growth model comparison. Let $\hat{Y}_{ij}^{k}$ be the fitted value obtained from model $k$, and $i = 1, \ldots ,215$, $j = 1, \ldots ,m_{i}$, and $k = 1, \ldots ,3$.

### Mean squared error (MSE)

**Fig. 1:**
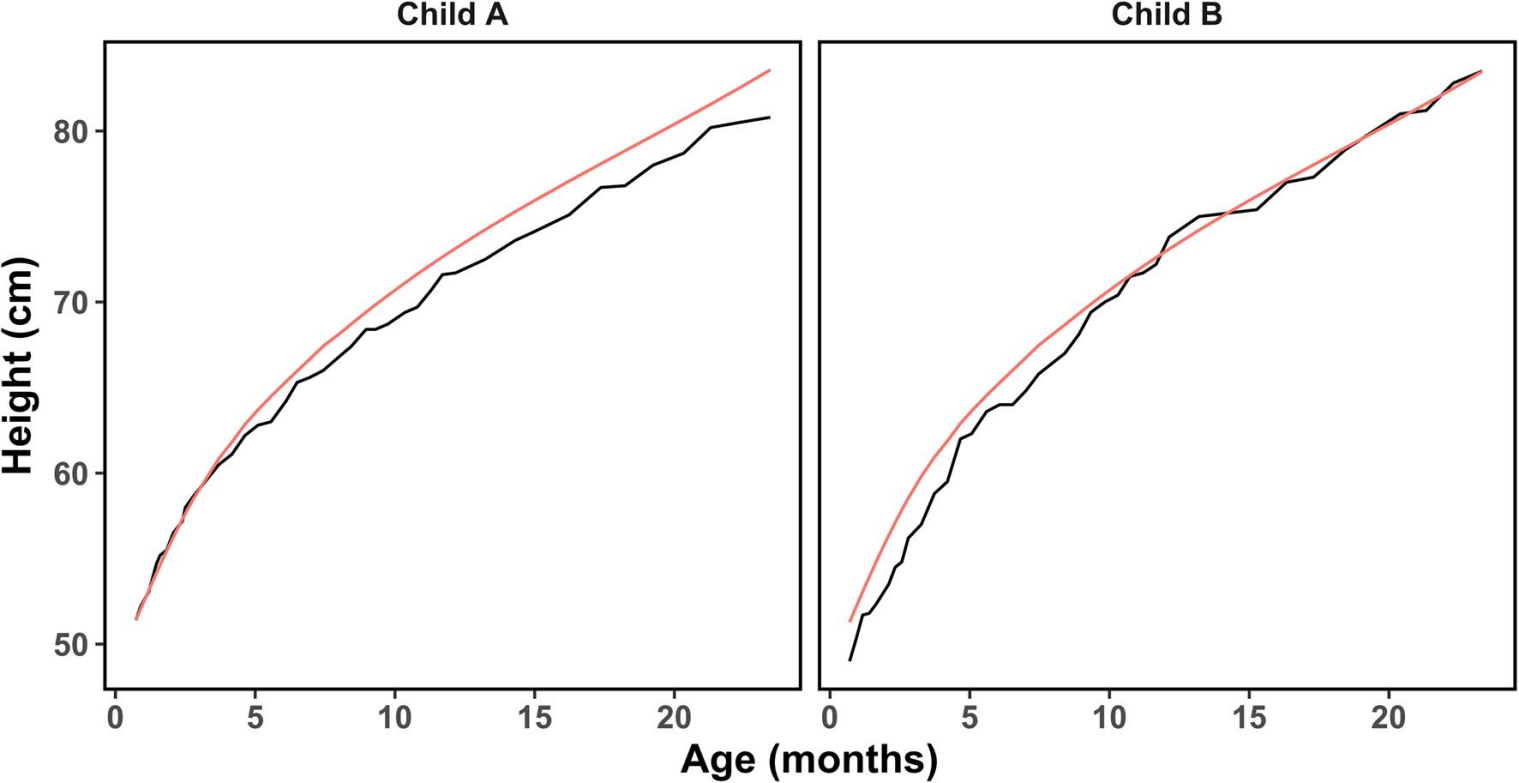
Age (x-axis) versus longitudinal height measurements (y-axis) showing fitted values (red) and observed values (black) for two separate individuals (child A and child B)

The first widely used selection metric is a subject specific mean squared error defined as

$$MSE_{i}^{k} = \frac{1}{{m_{i} }}\mathop \sum \limits_{j = 1}^{{m_{i} }} \left( {Y_{ij} - \hat{Y}_{ij}^{k} } \right)^{2}$$

where $i = 1, \ldots ,n$. Subject-specific MSEs can be combined to evaluate the performance of models on subpopulations of interest. One of the key limitations of using the MSE is demonstrated in Fig. 1. The black lines show observed growth curves of two selected children (child A and B), while the red lines show predicted growth curves. Child A has more error among larger height values, while child B tends to have increased error among smaller values. Un-normalized MSEs may disproportionately favor the most recent, almost always the largest, observations. As a result, the MSE for child A is inflated and greater than that of child B. However, normalization revealed that child A has lower overall error compared to child B. The scale of the data is not always consistent among subjects. Thus, subjects with larger measurements might dominate the comparisons when using metrics that contain original measure units such as MSE [44]. Moreover, subjects with larger changes can bring more difficulty in comparison when using MSE [45]. Despite these problems, practitioners and academicians still tend to rely this kind of absolute error measurement [44–46]. We next introduce three modifications to the MSE that better account for specifics of child growth data.

### Normalized mean squared error (nMSE)

It has been widely accepted that using relative error measurements which are unit-free can improve comparison performance and account for differences in measurement units as well as heteroscedasticity, thus providing fairer comparisons of predictive models [45, 47–49]. Subject-specific normalized mean squared error adds localized normalization and is defined as

$$nMSE_{i}^{k} = \frac{1}{{m_{i} }}\mathop \sum \limits_{j = 1}^{{m_{i} }} \frac{{\left( {Y_{ij} - \hat{Y}_{ij}^{k} } \right)^{2} }}{{Y_{ij}^{2} }}$$

which can be considered as percentage errors. The error expressed in percentages gives a more robust metric of goodness-of-fit that can be uniformly applied across a wide age span.

### Age-stratified mean squared error (aMSE)

Age-stratified mean squared error performs age-stratification and calculates within-strata subject-specific MSEs. It is defined as

$$aMSE_{is}^{k} = \frac{1}{{m_{i} }}\mathop \sum \limits_{{j \in Age_{s} }} \left( {Y_{ij} - \hat{Y}_{ij}^{k} } \right)^{2}$$

### Weighted mean squared error (wMSE)

It is also possible to create a metric using the MSE, nMSE, or aMSE that weights subgroups of interest. For example, we weighted individuals based on their growth velocity between 3 and 12 months so that slower growing individuals carried more weight. We used the following equations

$$\left( {Height\,\,velocity} \right)_{i} = \frac{{h_{ti12} - h_{ti3} }}{{t_{i12} - t_{ i3} }}$$

$$wMSE_{i}^{k} = \frac{1}{{m_{i} }}\mathop \sum \limits_{j = 1}^{{m_{i} }} \frac{{\left( {Y_{ij} - \hat{Y}_{ij}^{k} } \right)^{2} }}{{\left( { Quartile\,\,of\,\,height\,\,velocity} \right)_{i} }}$$

where $h_{ti}$ are height values at the corresponding $t_i$ (time points) closest to 3 and 12 months of age. We calculated quartile of height velocity based on each child’s growth velocity relative to all others in our sample. Therefore, the height velocities of children in the 0th–24th, 25th–49th, 50th–74th, and 75th–100th would be assigned values of 1, 2, 3, and 4, respectively. This is one example of weighting specific individuals; one could also weight other subject-specific metrics of interest (e.g. those with poorer outcomes).

### Model comparisons

**Fig. 2:**
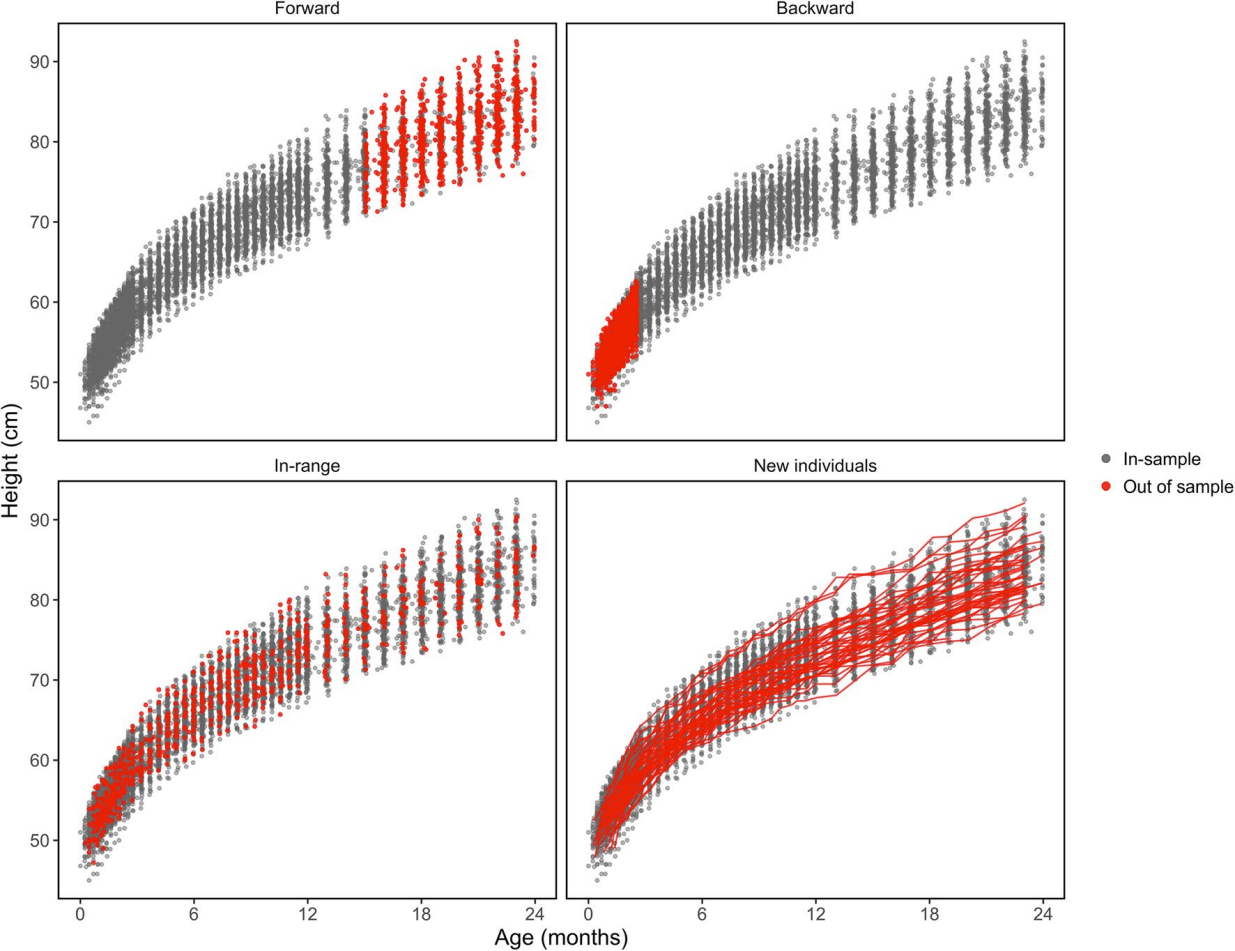
Age (x-axis) versus longitudinal height measurements (y-axis) stratified by those which were in-sample (used to fit regression models) versus out-of-sample (held out to make predictions on) and location of sampling. In-sample observations (grey) were used to fit models, while out-of-sample observations (red) were used to measure prediction accuracy

Four common scenarios in growth modeling were considered: forward, backward, in-range, and new individual prediction (Fig. 2). Forward prediction represents the scenario where missingness happens in the later stages of growth and the goal is to use data from the earlier stages to predict missingness in later stages. Backward prediction is opposite to forward; missingness in the early stages is predicted using data from later stages. In-range prediction happens where missingness takes place inside of the monitoring period, and new individual prediction occurs when there is missingness for an entire individual.

Error was measured by holding out a portion of the data (out-of-sample), fitting models to in-sample data, and then measuring predictive accuracy on the observations held out. With forward, backward, and in-range, analysis was performed by randomly selecting 50% of the children and subsequently holding out 10, 20, and 50% of their data. For new individual prediction, we randomly selected 10, 20, and 50% of the children to hold out. Primary analysis was performed using the 20% method, with the 10 and 50% used for comparison in sensitivity analysis.

Model performance will be presented as median and interquartile range (IQR) of MSE, nMSE, wMSE, or aMSE for each of the three model types (i.e. LME, fPCA, and FCR).

## Results

### Population characteristics

The final sample included 215 with complete data out of 304 eligible children. Eleven (3.6%) had incomplete anthropometric data and 78 (25.7%) did not have follow up past the age of 1 year. There were 39 observations per individual on average, with males representing 49% (n = 106) of the sample. Median lag between observations was 14 days (interquartile range 11–27).

### In-range

**Fig. 3:**
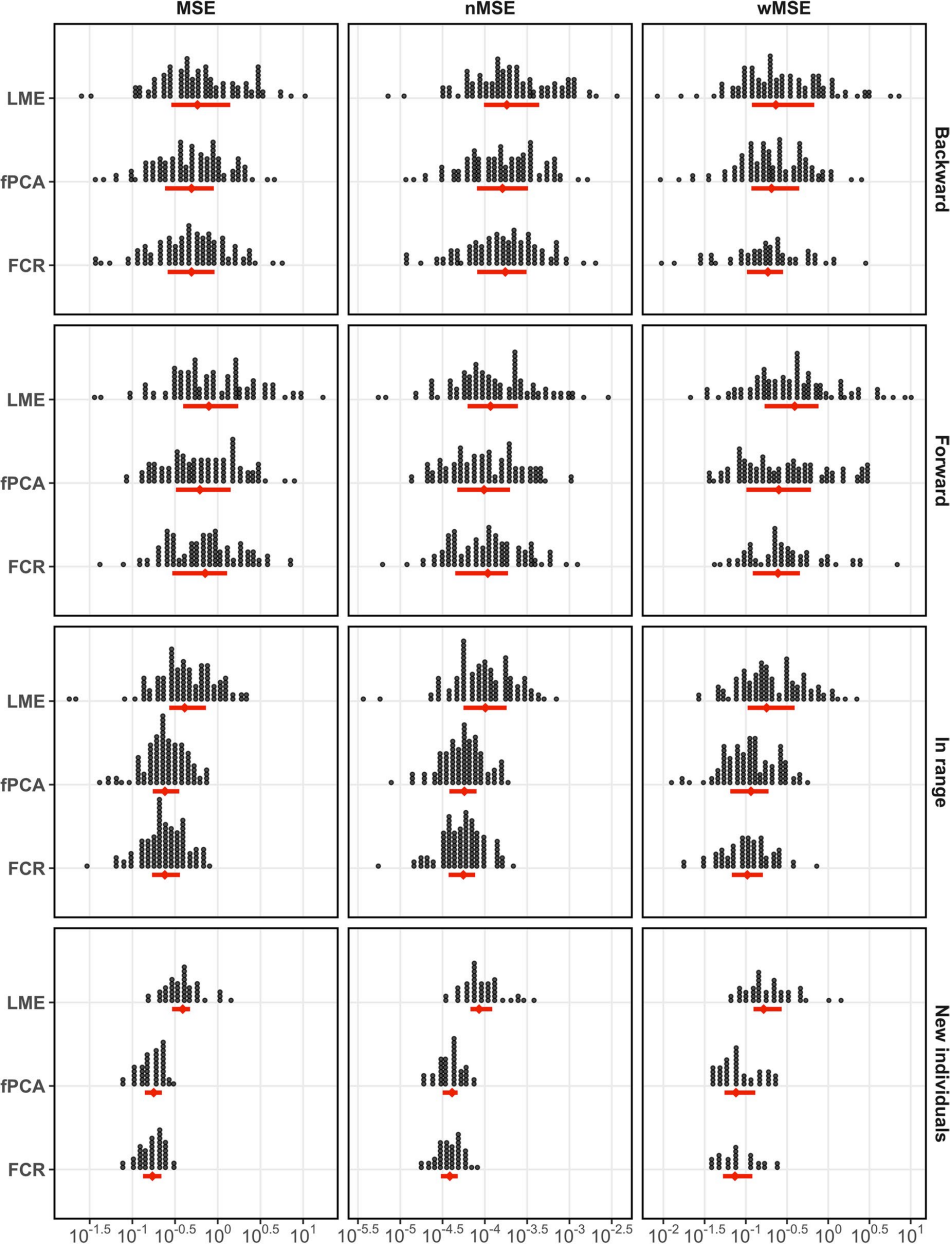
Prediction error (x-axis) versus model type (y-axis) stratified by prediction error metric (MSE, nMSE, and wMSE) and location of prediction (backward, forward, in-range, and new individuals). Median and interquartile range are presented in red (diamonds and error bars, respectively) and individual observations are presented in black (dots). The x-axis is log-scale with different ranges for MSE, nMSE, and wMSE due to differences in scale of prediction error values

**Fig. 4:**
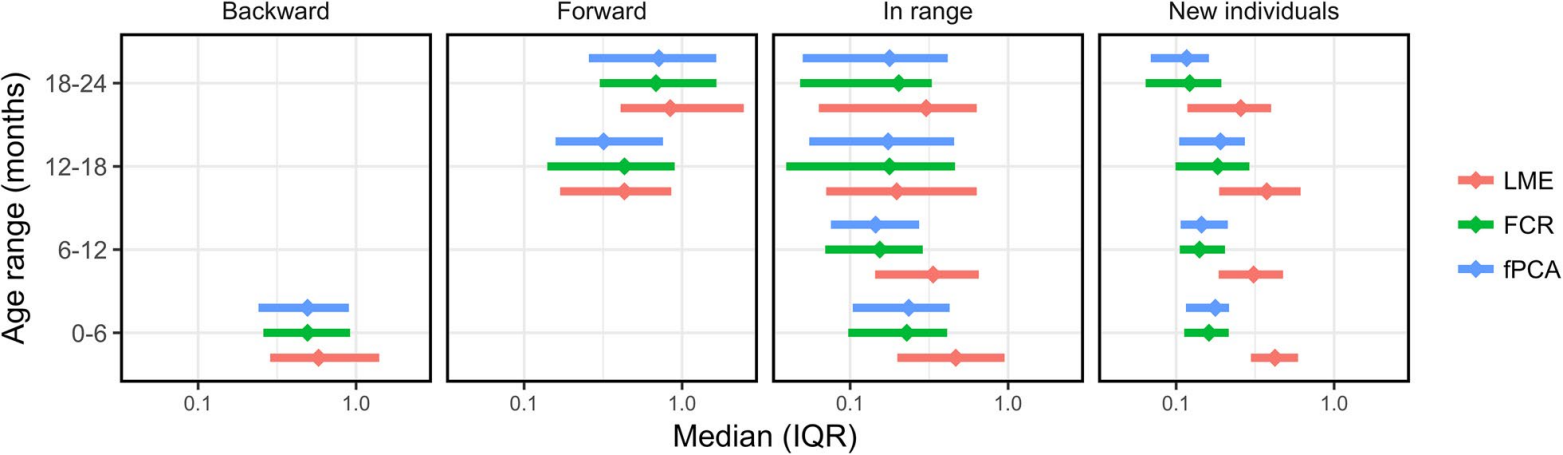
Results from age-stratified mean squared error (aMSE). MSE values (x-axis) versus age-group (y-axis) stratified by location of prediction (backward, forward, in-range, and new individuals) and model type (LME, FCR, and fPCA). Median values are presented as diamonds and interquartile ranges as error bars

In-range prediction error was lowest with fPCA when using nMSE, lowest with FCR when using wMSE, and the same when using MSE (Tables 1, 2, and 3). Median nMSE ranged from 10.1E−5 (IQR 5.6E−5 to 18.1E−5) for LME to 5.6E−5 (3.7E−5 to 7.7E−1) for FCR. Distributional properties of prediction error for each model, metric, and sampling method can be seen in Fig. 3. Using aMSE, we saw varying distributions again with in-range prediction. FCR and fPCA performed similarly well followed by LME (Fig. 4).

**Table 1:** Median and interquartile range for MSE stratified by location of prediction and model type

	Backward	Forward	In-range	New individuals
LME	0.58 (0.29, 1.40)	0.79 (0.39, 1.74)	0.41 (0.27, 0.73)	0.39 (0.29, 0.47)
FCR	0.49 (0.26, 0.92)	0.71 (0.29, 1.29)	*0.23 (0.17, 0.36)*	*0.17 (0.13, 0.22)*
fPCA	*0.48 (0.24, 0.90)*	*0.62 (0.32, 1.42)*	0.24 (0.17, 0.35)	0.18 (0.14, 0.22)

Best performing models are in italics. Error was measured by holding out a portion of the data (out-of-sample), fitting models to in-sample data, and then measuring predictive accuracy on the observations held out. With forward, backward, and in-range, analyses were performed by randomly selecting 50% of the children and subsequently holding out 20% of their data

**Table 2:** Median and interquartile range for nMSE, stratified by location of prediction and model type

	Backward	Forward	In-range	New individuals
LME	18.19 (9.77, 43.90)	11.67 (6.25, 24.59)	10.14 (5.59, 18.08)	8.55 (6.75, 12.21)
FCR	17.44 (8.11, 31.11)	10.84 (4.47, 18.78)	*5.57 (3.73, 7.67)*	*3.85 (3.02, 4.78)*
fPCA	*16.17 (8.05, 32.35)*	*9.78 (4.72, 19.86)*	5.72 (3.81, 7.98)	4.10 (3.17, 4.76)

Best performing models are in italics. All values in Table 2 were multiplied by 10^5^ to help better visualize performance differences. Error was measured by holding out a portion of the data (out-of-sample), fitting models to in-sample data, and then measuring predictive accuracy on the observations held out. With forward, backward, and in-range, analyses were performed by randomly selecting 50% of the children and subsequently holding out 20% of their data

**Table 3:** Median and interquartile range for wMSE, stratified by location of prediction and model type

	Backward	Forward	In-range	New individuals
LME	0.23 (0.12, 0.68)	0.39 (0.17, 0.76)	0.18 (0.11, 0.39)	0.17 (0.12, 0.27)
FCR	*0.19 (0.10, 0.28)*	*0.24 (0.12, 0.45)*	*0.10 (0.06, 0.16)*	*0.07 (0.05, 0.12)*
fPCA	0.21 (0.12, 0.45)	0.25 (0.10, 0.62)	0.12 (0.07, 0.19)	0.08 (0.06, 0.13)

Best performing models are in italics. Error was measured by holding out a portion of the data (out-of-sample), fitting models to in-sample data, and then measuring predictive accuracy on the observations held out. With forward, backward, and in-range, analyses were performed by randomly selecting 50% of the children and subsequently holding out 20% of their data

### Forward

Model error with forward prediction, using median nMSE, ranged from 0.79 (IQR 0.39–1.74) for LME to 0.62 (0.32–1.42) for fPCA. Model error was lowest for fPCA when utilizing MSE and nMSE, but FCR slightly outperformed fPCA with wMSE (Tables 1, 2, and 3). There were similar results using aMSE with fPCA performing best, followed by FCR and LME (Fig. 4). Intra-strata comparison showed a trend, with FCR, fPCA, and LME performing better in ages 12–18 months compared to ages 18–24 months.

### Backward

Backward prediction revealed similar results. fPCA performed best using MSE and nMSE, but FCR performed best with wMSE (Tables 1, 2, and 3). Median nMSE ranged from 18E−5 (IQR 9.8E−5 to 43.9E−5) for LME to 16.2E−5 (8.1E−5 to 32.3E−5) for fPCA. All predicted points in backward prediction fell between ages 0–6 months. Therefore, the aMSE did not stratify the data and was interpreted as the standard MSE.

### New individuals

When predicting in-range on new individuals, FCR slightly outperformed fPCA for all metrics and sampling methods. Median nMSE ranged from 8.6E−5 (IQR 6.6E−5 to 12.2E−5) for LME to 3.9E−5 (3.0E−5 to 4.8E−5) for FCR. Error distributions using aMSE were consistent with the above findings, with FCR and fPCA performing best followed by LME (Fig. 4). Between-strata differences were more apparent for LME, with LME showing less error at higher age ranges.

### Sensitivity analyses

**Fig. 5:**
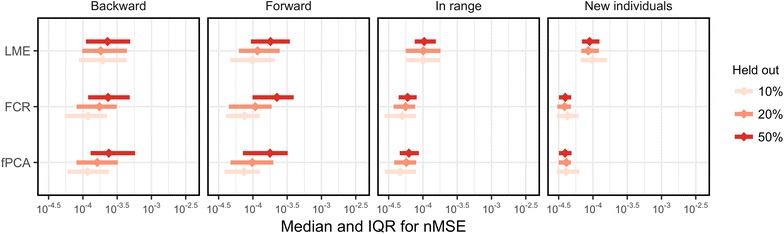
Prediction error (x-axis) versus model type (y-axis) stratified by location of prediction (backward, forward, in-range, and new individuals). Median and interquartile range are presented in red (diamonds and error bars, respectively). The x-axis is log-scale

As seen in Fig. 5, prediction error in backward, forward, and in-range tended to be larger with increased number of observations held out. This trend was not as apparent when predicting on new individuals.

## Discussion

This analysis demonstrates how to compare growth models (both nested and non-nested) by measuring prediction error via nMSE, wMSE, and aMSE. Each metric is subject-specific and can be used in a variety of real world situations. Sampling techniques can be adjusted to replicate exact scenarios of interest. Utilizing the nMSE and aMSE addresses the issue of the MSE favoring larger measurements. Furthermore, the aMSE can illuminate intra-age group performance differences and the wMSE demonstrates the ability to weight specific subgroups of interest, potentially helping to further detect performance gaps between growth models.

Based on the results of this study, functional models outperformed traditional linear models in all scenarios. Even when utilizing proven techniques with LME (i.e. truncated cubic splines and autoregressive correlation correction), FCR and fPCA performed better in all scenarios tested [21, 50]. The difference in prediction error between FCR and fPCA in most situations was relatively small. Employing the wMSE revealed a shift in the best performing model when predicting backward, forward, and in-range. In these situations, the MSE and nMSE preferred fPCA as the best performing model while the wMSE showed FCR outperforming fPCA (Tables 1, 2, and 3). While functional models consistently outperformed LME, it seems they were more sensitive to the proportion of data removed when predicting backward and in-range (Fig. 5). One possible explanation is that shapes of curves are well defined for LME with cubic splines; however, for functional approaches, it is more difficult to predict trajectories of growth curves with limited amount of data.

A limitation of the MSE is its tendency to be inflated by outliers. Using subject-specific estimates partially addresses this, but there is still the possibility of having outliers within subjects. Sensitivity analysis should be performed to assess whether more robust (outlier-insensitive) approaches are necessary. There are a few other limitations to this study. First, even though we used a variety of sampling strategies, they do not comprehensively represent real world situations. There are more scenarios that could not be included in this analysis, such as predicting backward and forward on new individuals as well as choosing different hold out percentages. Second, aMSE can be less useful in certain situations. For example, age-stratification may not be needed when predicting over a relatively short age range or if data is sparse with fewer observations in each age group.

Our study also has some potential strengths. First, the proposed method is a novel approach of transforming the subject-specific MSE (i.e. nMSE, aMSE, and wMSE) to assess prediction error differences between both nested and non-nested growth models. Alternative methods such as AIC, BIC, F-test, and the LRT only work for nested models. Second, our approach is flexible, allowing adaptation to specific real-world situations. The ability to weight subgroups of interest and adapt the age ranges used with aMSE contributes to this. Third, the CONTENT dataset is of high quality and high resolution. There were very few outliers regarding growth trends and the average number of observations per child was approximately 40 within a 2-year span. Finally, this analysis employed modern growth modeling techniques. FCR, fPCA, and LME are proven effective techniques for longitudinal growth modeling [13, 22, 24, 26–29, 32–39, 51–53].

## Conclusion

Subject-specific normalized mean squared error, age-stratified mean squared error, and weighted mean squared error are useful metrics for comparing both nested and non-nested growth models. We applied these metrics to three competing modeling methods and demonstrated the ability to weight subgroups of interest and evaluate performance gaps.
